# Evaluation of Medicare Coverage and Estimated Out-of-Pocket Costs for Generic Abiraterone Products

**DOI:** 10.1001/jamanetworkopen.2022.31475

**Published:** 2022-09-30

**Authors:** Arjun Gupta, Emmanuel S. Antonarakis, Anne H. Blaes, Christopher M. Booth, Stacie B. Dusetzina

**Affiliations:** 1 Division of Hematology, Oncology, and Transplantation, University of Minnesota, Minneapolis; 2 Department of Oncology, Queen’s University, Kingston, Canada; 3 Cancer Care and Epidemiology, Queen’s University, Kingston, Canada; 4 Department of Health Policy, Vanderbilt University Medical Center, Nashville, Tennessee; 5 Vanderbilt-Ingram Cancer Center, Nashville, Tennessee

## Abstract

This cross-sectional study evaluates the association of generic competition and changes in product coverage and cost-sharing by using Medicare data and estimated out-of-pocket costs for the oral specialty drug abiraterone.

## Introduction

Generic competition lowers drug prices^1^ but may not reduce out-of-pocket costs for patients.^2^ To better understand the association between generic competition and changes in product coverage and cost sharing, we examined abiraterone, an oral specialty drug that was first approved by the US Food and Drug Administration (FDA) in 2011 and is commonly used to treat prostate cancer. Multiple recent generic market entrants across 2 commonly used dosage units (250 and 500 mg) make abiraterone ideal for evaluating these associations.

## Methods

 The University of Minnesota deemed this cross-sectional study exempt from review, and informed consent was waived because we used deidentified data. The study followed the STROBE reporting guideline.

We studied 4 abiraterone products, including 250- and 500-mg generic and brand-name tablets, for a common total dosage of 1000 mg/d. We used the FDA Orange Book to identify market entry of generic products approved through May 2022. We used Medicare Prescription Drug Plan Formulary and Pricing Information Files (2018-2022) to identify product coverage and expected annual out-of-pocket costs.^3,4^ We calculated point-of-sale prices (excluding rebates and discounts) and out-of-pocket costs assuming monthly fills, standard plan benefit design, and no other medication use. We also calculated a best-case scenario of 2022 out-of-pocket costs (lowest-cost Part D plan/pharmacy combination in Minneapolis, Minnesota) using the Medicare Part D Plan Finder,^5^ assuming the patient was diagnosed in January 2022. We analyzed data using Excel version 16.61 (Microsoft).

## Results

**Figure 1.**
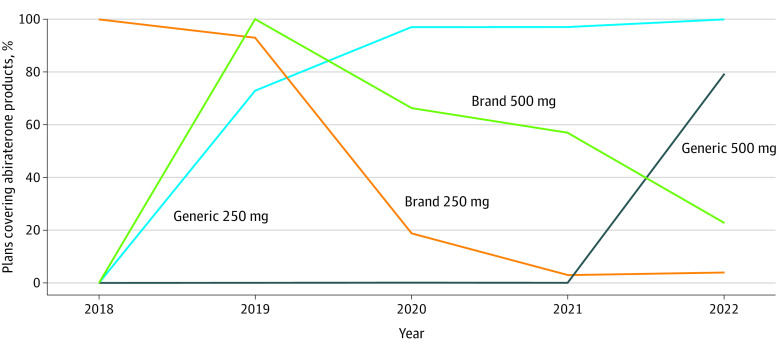
Percentage of Medicare Part D Prescription Drug Plans Covering 250- and 500-mg Generic and Brand-Name Abiraterone Products from 2018 to 2022

The proportion of Medicare Part D plans covering generic abiraterone increased from 0% for the 250- and 500-mg formulations in 2018 to 100% and 79%, respectively, in 2022 (Figure 1). Between 2018 and 2022, 12 generic 250-mg and 4 generic 500-mg abiraterone formulations came to market, with the first available in October 2018 and December 2020, respectively.

**Figure 2.**
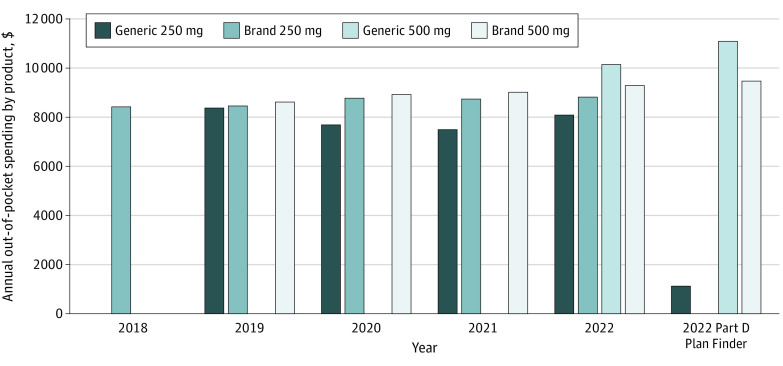
Estimated Annual Out-of-Pocket Costs for Different Abiraterone Products (1000-mg/d Total Dose) From 2018 to 2022 The Medicare Part D Plan Finder price assumes no low-income subsidies, no other medication use, and use of the cheapest plan/pharmacy combination in Minneapolis, Minnesota. The 2022 Part D Plan Finder price for the 250-mg brand-name product is not shown because it was greater than $150 000; this reflects its lack of coverage by most Part D plans.

Expected annual out-of-pocket costs ranged from $7491 to $10 148 across products and years (Figure 2). In 2022, out-of-pocket costs for 500-mg generic and brand-name abiraterone were $10 148 and $9298, respectively. The same was true when the Part D Plan Finder was used (Figure 2). However, out-of-pocket costs were much higher for 250-mg brand-name vs generic abiraterone (>$150 000 vs $1139).

## Discussion

Despite preferred coverage over brand-name products on Medicare Part D formularies, generic abiraterone was associated with modest savings for beneficiaries in this cross-sectional study. In fact, out-of-pocket costs were higher for the 500-mg generic vs brand-name products in 2022. A patient able to price shop could pay approximately $1100 for a 1-year supply of 250-mg generic abiraterone—one-seventh of the mean annual out-of-pocket costs.

This study has several key findings. First, although plans rapidly covered generic products, reductions in out-of-pocket costs were delayed. The 250-mg generic product (available since 2018, with 12 manufacturers) is now less costly than its brand-name counterpart, but the 500-mg product is not. It took more than 3 years for costs to decrease, mirroring data reporting that it takes multiple years and/or entrants for robust price competition.^1,4^ Second, out-of-pocket costs for Part D beneficiaries can be higher with generic products despite lower list prices (vs brand-name products) because of a program design flaw.^2,4^ Finally, we observed a broad range of out-of-pocket costs for the same drug and/or dose. Emphasizing this point, a patient without insurance can obtain an annual supply of abiraterone for $2202 from an online pharmacy platform—lower than through their health plan.^6^

A limitation of this study is that Medicare formulary files capture drug prices from the prior 3 months and may not reflect current prices. Regardless, Medicare Part D beneficiaries do not immediately benefit from price reductions associated with generic entry for specialty drugs. Proposals to correct the flaw that makes high-cost generic drugs more expensive to patients than brand-name drugs include eliminating manufacturer discounts from out-of-pocket spending calculations for brand-name drugs or extending the discount to generics. Alternatively, recent proposals to redesign Medicare Part D eliminate the coverage gap and cap out-of-pocket spending, which would avoid penalizing beneficiaries for generic drug use.
